# Trigger Finger Caused by a Solitary Osteochondroma of the Proximal Phalanx in an Adolescent: A Case Report

**DOI:** 10.7759/cureus.79691

**Published:** 2025-02-26

**Authors:** Hirotaka Kurata, Michiyuki Hakozaki, Shinichirou Yoshida, Jun Iwatsu, Toshitake Aizawa

**Affiliations:** 1 Department of Orthopaedic Surgery, Tohoku University Hospital, Sendai, JPN; 2 Higashi-Shirakawa Orthopaedic Academy, Fukushima Medical University School of Medicine, Fukushima, JPN; 3 Department of Orthopaedic Surgery, Fukushima Medical University School of Medicine, Fukushima, JPN; 4 Department of Orthopaedic Surgery, Iwaki City Medical Center, Iwaki, JPN

**Keywords:** proximal phalanx, solitary osteochondroma, stenosing tenosynovitis, trigger finger, tumorous lesions

## Abstract

Trigger finger is a common disease, and stenosing tenosynovitis is the most frequent cause of this condition in middle-aged women. We report a case of surgical excision in a 16-year-old adolescent male with trigger finger symptoms caused by a solitary osteochondroma at the proximal end of the proximal phalanx. Although tumorous lesions are a rare cause of trigger finger, a differential diagnosis is necessary because the treatment strategy and surgical technique employed to treat this condition differ from those employed for common causes, such as stenosing tenosynovitis and pediatric trigger thumb.

## Introduction

Although trigger finger is a common condition in women after middle age, it is uncommon in adolescents [1]. On the other hand, solitary osteochondroma is a common and benign bone tumor that generally occurs around the knee and stops growing as the growth plates close [2]. However, its occurrence in the hand is rare. Here, we report a case of adolescent-onset trigger finger due to osteochondroma of the proximal phalanx of the right middle finger.

## Case presentation

The patient was a 16-year-old adolescent male with no remarkable previous medical history and no notable family history, including hereditary multiple osteochondromatosis. One year prior to his first visit to our hospital, he became aware of a tugging sensation on both flexion and extension of his right middle finger. The symptoms gradually worsened and led to a trigger finger. He visited a nearby clinic and was diagnosed with a trigger finger caused by a bony prominence from the palpation and radiological findings. Then, he was referred to our hospital for the purpose of surgical treatment. Although his sports history included Karate from the age of nine to 11 years, and volleyball from the age of 12 to 15 years, there was no history of blunt trauma to the flexor tendons.

The bony prominence was palpated on the palmar side of the metacarpophalangeal (MP) joint of the right middle finger without tenderness, and snapping was observed when extending the finger from the position of maximum flexion (Video 1). There were no other bony prominences on the whole body.

**Video 1:** Trigger finger observed by the first author.

**Figure 1:**
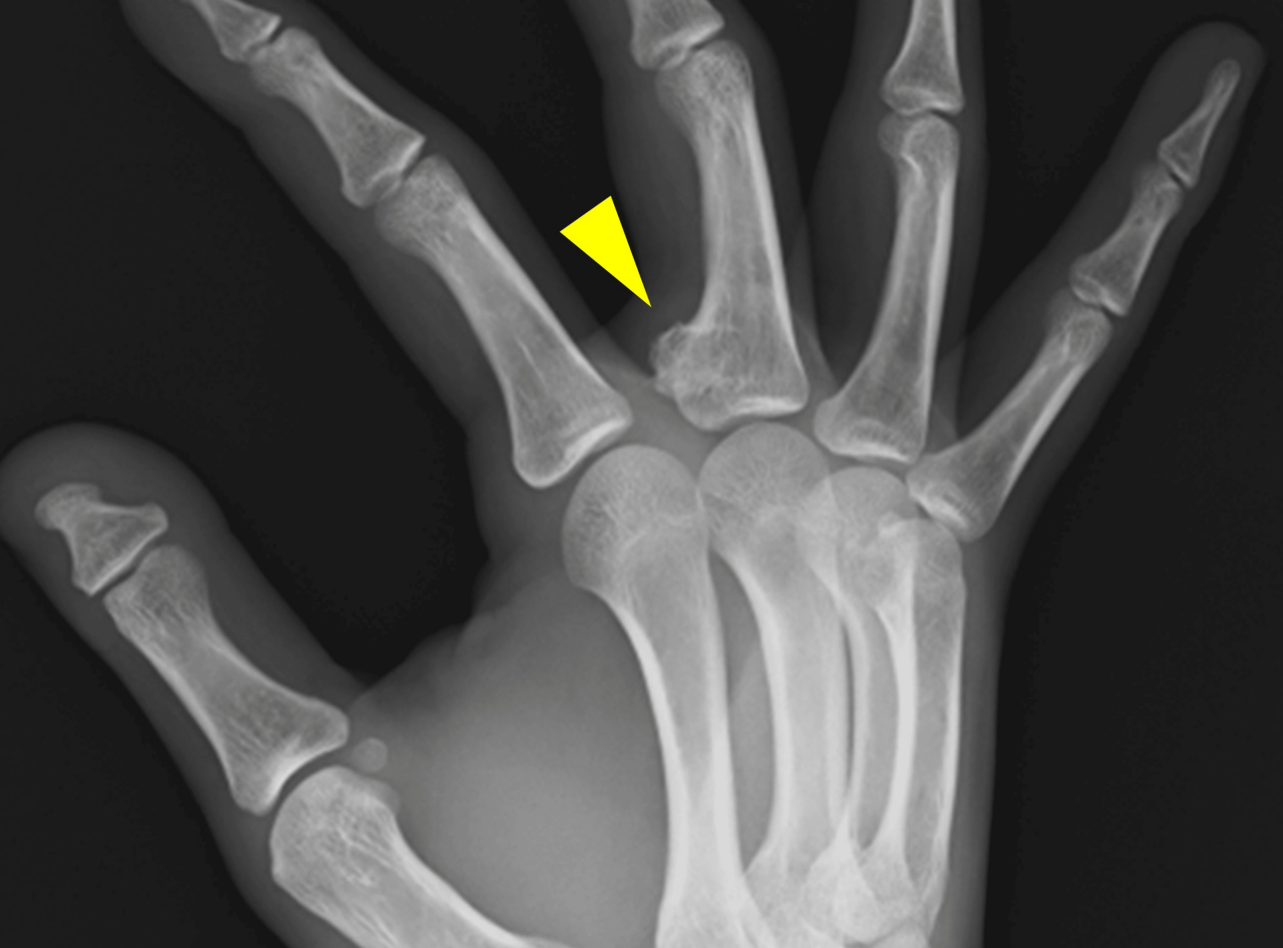
Plain radiograph. A bony prominence on the palmar side of the proximal end of the middle phalanx is visible.

Plain radiographs indicated a bony prominence on the palmar side of the proximal end of the middle phalanx (Figure 1).

**Figure 2:**
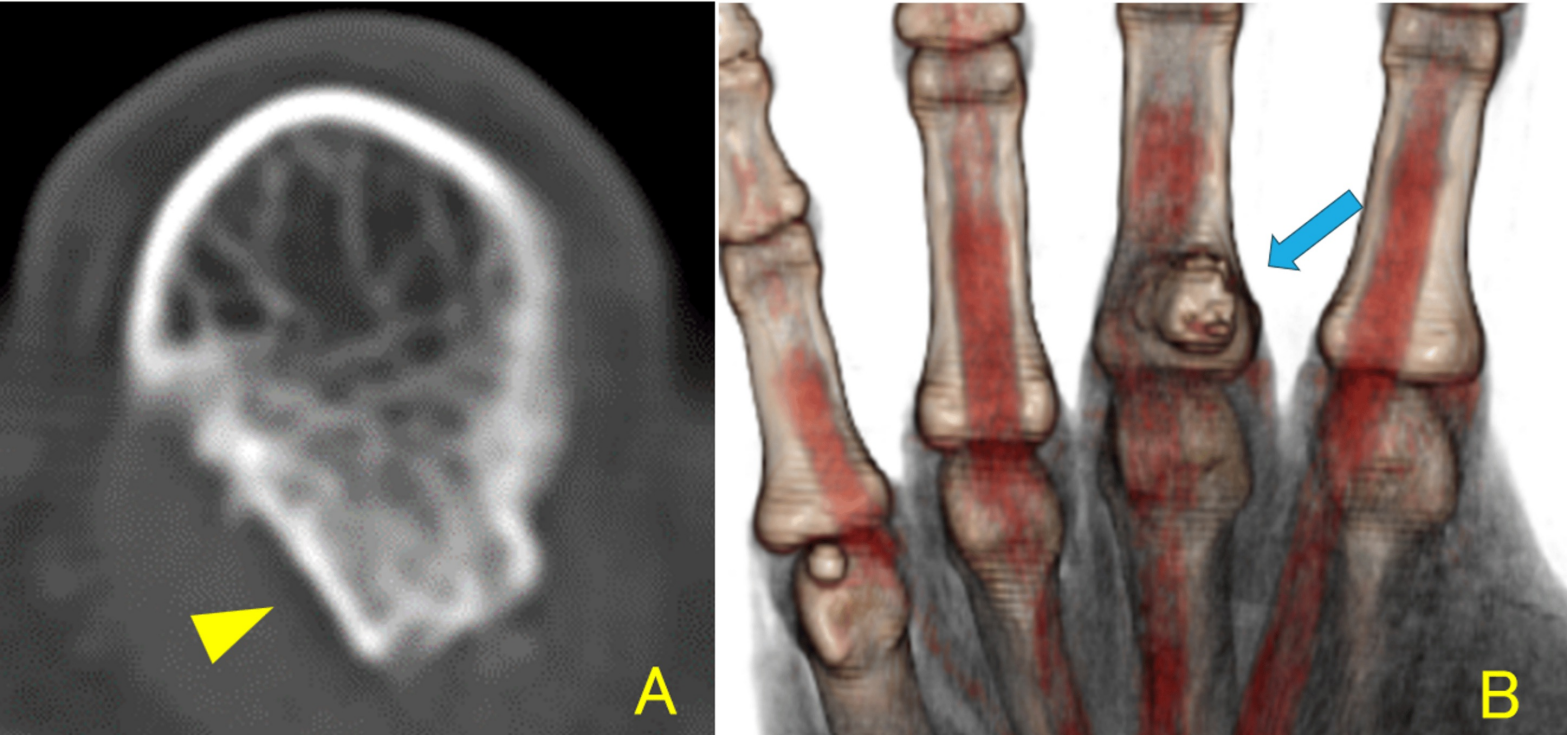
Computed tomography. (A) A 13 × 10 × 9 mm-sized bony prominence in continuity with the bone marrow is visible. (B) Ulnar deviation of the flexor tendon is visible.

Computed tomography (CT) revealed a 13 × 10 × 9 mm-sized bony prominence in continuity with the bone marrow and ulnar deviation of the flexor tendon (Figure 2).

**Figure 3:**
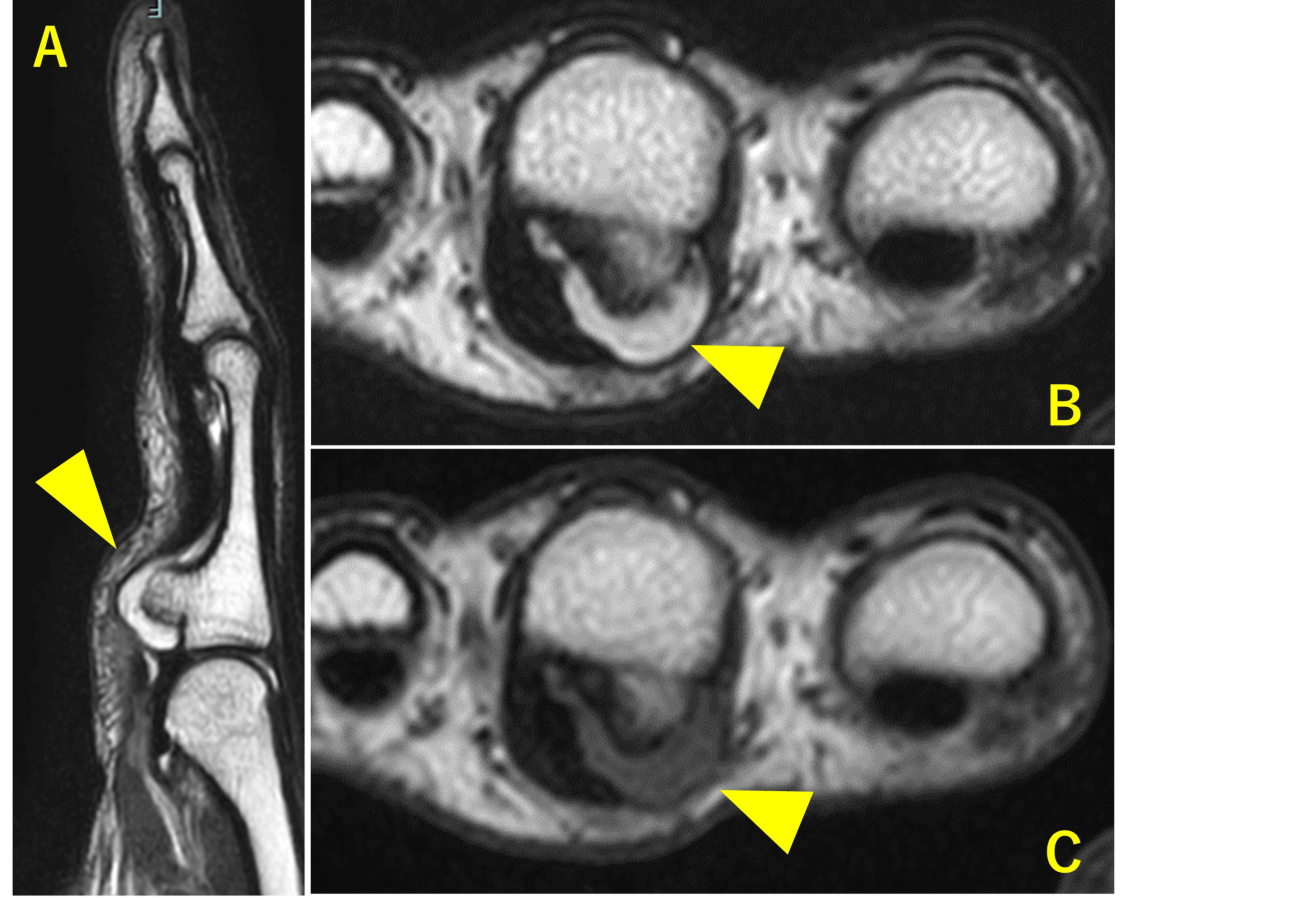
Magnetic resonance imaging. (A) T2-weighted imaging, sagittal view. (B) T2-weighted imaging, axial view. (C) T1-weighted imaging, axial view. Bone marrow continuity and a thin cartilage cap covering the bony prominence are visible.

Magnetic resonance imaging (MRI) showed bone marrow continuity and a thin cartilage cap covering the bony prominence (Figure 3).

The patient was diagnosed with a trigger finger caused by osteochondroma, and we decided to perform surgical resection of the tumor.

**Figure 4:**
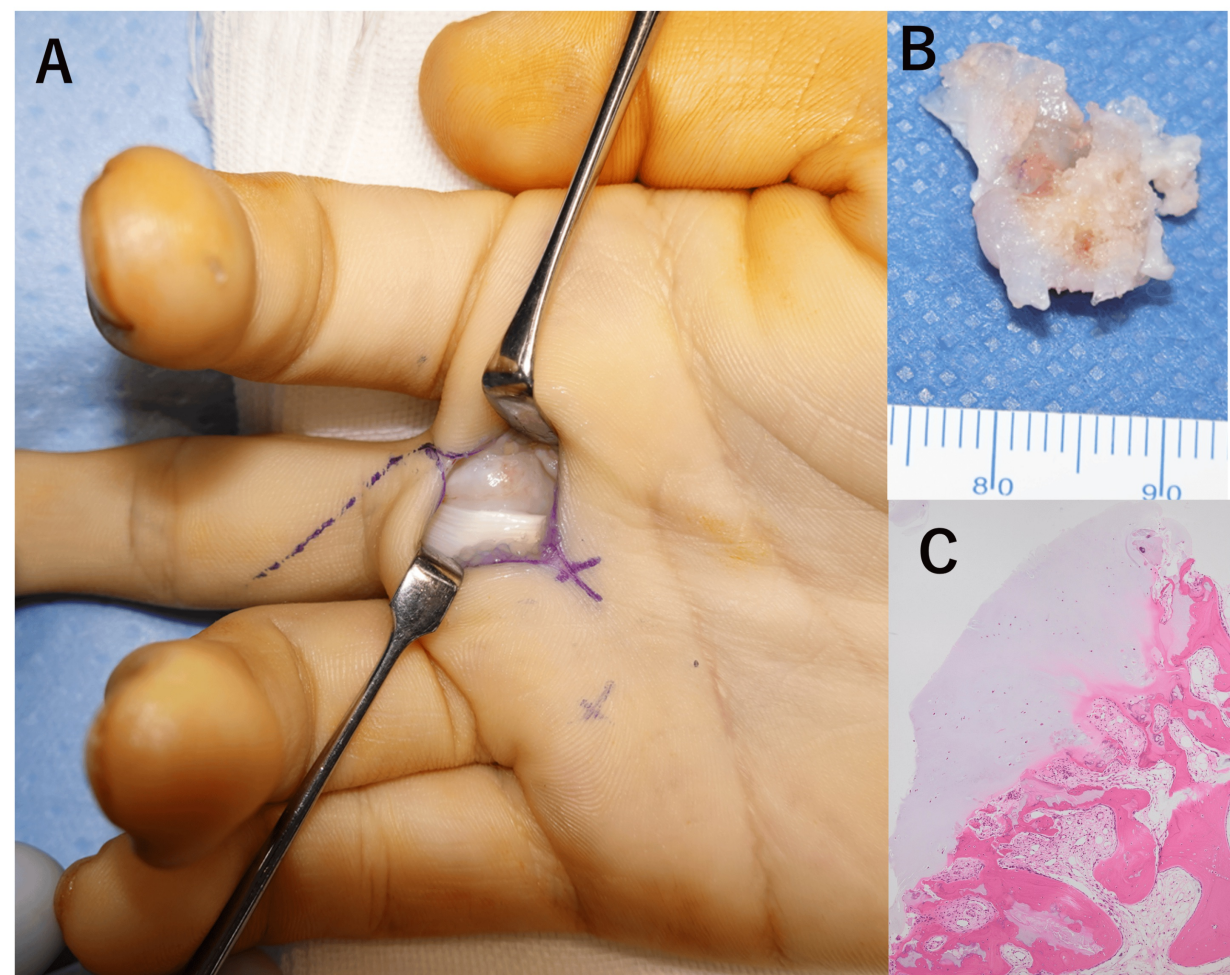
Intraoperative photographs of the patient's hand and the resected tumor, and a histological section of the surgical specimen. (A) The flexor digitorum profundus and superficialis tendons were intact, and the tumor caused an ulnar deviation and elongation of the tendons. (B) Resected tumor. (C) Microscopically, a cartilage cap covering the bony prominence and bone marrow continuity are visible (hematoxylin and eosin staining).

At the time of surgery, gross findings presented no thickening or inflammation in the A1 pulley. Both the flexor digitorum profundus and superficialis tendons were intact, and the tumor caused an ulnar deviation and elongation of the tendons (Figure 4).

The tumor surface was covered with a white cartilaginous cap. Excision of the bony prominence resulted in the exposure of the marrow cavity and the snapping phenomenon vanished. Pathological findings of the excised tumor revealed bone marrow continuity and cartilage cap coverage, and the tumor was diagnosed on the basis of histopathologic examination as an osteochondroma. There was no recurrence of the trigger finger at three months postoperatively.

## Discussion

The occurrence of a trigger finger shows a bimodal peak in childhood and middle age. The most common cause of trigger finger is stenosing tenosynovitis of the A1 pulley in adults, followed by stiff thumb due to Notta’s node in children, which is also known as pediatric trigger thumb [1].

Stenosing tenosynovitis most frequently occurs between the fifth and sixth decades of life in women, who are affected up to six times more than men. Patients with diabetes mellitus, carpal tunnel syndrome, de Quervain’s disease, hypothyroidism, rheumatoid arthritis, renal disease, and amyloidosis are at a greater risk for trigger finger development. The ring finger is most commonly affected, followed by the thumb (trigger thumb), middle, index, and small fingers in patients with multiple trigger digits [3].

Various treatment methods have been established as conservative treatments for trigger fingers due to stenosing tenosynovitis. It is estimated that orthotic treatment improves symptoms in 50% to 90% of patients. There are numerous protocols, including all-day immobilization of the MP joint in mild flexion at approximately 15° (aluminum splint, plaster splint, or taping) for approximately six weeks [4]. Intra-tendinous steroid injection is the most common conservative treatment because of its high efficacy in the early stage. However, tendon rupture is reported as a serious side effect of steroid injection, in particular, for patients with type 1 diabetes; hence, surgery may be the first-line treatment for such patients. Surgery is the treatment of choice in cases refractory to conservative treatment [5]. Stenosing tenosynovitis in the A2 pulley may be recognized by an elastic phenomenon that does not disappear after the incision of the A1 pulley. It can be differentiated when the A1 pulley and the flexor tendon are not hypertrophied while the A2 pulley is. Ultrasonographic examination is useful in such cases, being non-invasive and cost-effective [6].

Pediatric trigger thumb may develop during infancy since many cases are not noted during neonatal examinations [7]. Some clinicians recommend splinting the thumb in full extension and the reported rate of spontaneous relief from the flexed posture varies from 0% to 49%. A1 pulley release after age three is the definitive surgical treatment [8].

On the other hand, a trigger finger due to a bone tumor is extremely rare. Schwaiger et al. reported a case of trigger finger due to an extraosseous chondroma that resolved after surgical excision, and they found that ultrasonographic examination was useful in the diagnosis [9]. Al-Harthy and Rayan reported a case of trigger finger caused by osteochondroma of the basal phalanx in a five-year-old girl, whose tumor had not been detected until intraoperative observation [10].

Trigger finger caused by an osteochondroma originating from the phalanges is extremely rare. While reports of pediatric trigger fingers due to multiple osteochondromas are relatively common, to our knowledge, only three cases (including the present case) of trigger fingers caused by a solitary osteochondroma originating from the proximal phalanx have been reported (Table 1) [10,11].

**Table 1:** Previously reported cases of trigger finger caused by solitary osteochondroma of the proximal phalanx.

Authors	Publication year	Age (years)	Sex	Location	Illness duration	Therapy
Al-Harthy and Rayan [10]	2003	5	Woman	Right ring finger	9 month	Surgical excision
Kwon and Kang [11]	2020	21	Man	Left middle finger	2 month	Surgical excision
Our case	2025	16	Man	Right middle finger	12 month	Surgical excision

In multiple osteochondromatosis, previous studies have indicated that hand osteochondroma was primarily asymptomatic and rarely required surgical intervention. Only two of 32 patients in the study by Komura et al. required surgical treatment, including exostectomy for correcting impingement on the flexor tendons and osteotomy for angulation [12]. In this case, the palpable bony prominence suggested a space-occupying lesion and preoperative examinations indicated that the lesion was an osteochondroma, leading to the decision for its excision.

Differential diagnoses of space-occupying lesions of the hand and finger, ranging from benign tumors such as a giant cell tumor of the tendon sheath, fibroma of the tendon sheath, and ganglion cysts, to malignant tumors such as synovial sarcoma, should be considered. In the case of trigger finger caused by abnormal tendon function due to an occupying lesion (as in the present case), conservative treatment for stenosing tenosynovitis is not expected to be effective, and imprudent conservative treatment without ruling out a differential diagnosis is time-consuming and costly for the patient. It is crucial to enhance diagnostic accuracy with preoperative MRI and other imaging techniques to avoid unfortunate outcomes such as an unplanned excision. Especially in cases like the present case, where the patient is not of the age when a trigger finger is most likely to occur, diagnosis and treatment should be made with the possibility of a tumor in mind to avoid inadvertent treatment.

Trigger finger caused by solitary osteochondroma of the hand and finger is an extremely rare condition with a limited number of cases, with only one case reported in the present study and only two cases reported in the past. Future accumulation of cases is needed for a more detailed study of the pathogenesis and appropriate treatment, which is a limitation of the present study.

## Conclusions

We have reported an extremely rare case of adolescent-onset trigger finger due to osteochondroma of the proximal phalanx of the right middle finger, which was surgically treated. When diagnosing trigger fingers in young patients, especially in fingers other than the thumb, it is important to consider the possibility of tumorous lesions, including osteochondroma of the phalanges, as a potential cause. Imaging evaluations such as plain radiography and ultrasonography are useful. Since conservative treatment may be ineffective and surgical methods vary, it is essential to distinguish tumorous lesions from stenosing tenosynovitis.
